# Pentraxin 3 levels from bronchoalveolar lavage of critically ill patients predict lung infection

**DOI:** 10.1186/cc9699

**Published:** 2011-03-11

**Authors:** T Mauri, A Pradella, A Confalonieri, G Bellani, D Ferlicca, M Bombino, I Cuccovillo, N Patroniti, A Mantovani, A Pesenti

**Affiliations:** 1 Universita degli Studi di Milano-Bicocca, Monza, Italy; 2 San Gerardo Hospital, Monza, Italy; 3 Humanitas Clinical Institute, Rozzano, Italy

## Introduction

Timely diagnosis of lung infection in critically ill patients is key to guide therapy and avoid futile antibiotic prescription. The gold standard for diagnosis is microbiological culture of bronchoalveolar lavage fluid (BALf). However, it takes up to 48 hours to disclose results. Pentraxin 3 (PTX3) is an acute phase mediator of infection that can be assayed in a few hours. We described a relationship between BALf PTX3 presence and lung infection in acute respiratory distress syndrome patients. The aim of this study was to validate BALf PTX3 as an early marker of lung infection in critically ill patients.

## Methods

We collected 40 consecutive BALfs from 36 adult patients admitted to our general ICU. BALfs were collected by standard technique and cultured when lung infection was clinically suspected (that is, pulmonary infiltrate + presence of fever, leukocytosis or leukopenia and purulent secretions). We collected plasma samples at the same time as BALf sampling. We assayed PTX3 in BALf and plasma by ELISA (detection limit 0.1 ng/ml) and we recorded BALf microbiology results. We defined lung infection when noncontaminant microbe was identified in ≥10^4 ^cfu/ml. Analyses were performed by simple regression, chi-square or Fisher exact test and ROC curve analysis, as appropriate.

## Results

Lung infection was diagnosed in 14/40 cases (35%). Three out of 14 (21%) were defined as community-acquired pneumonia, 4/14 (28%) were hospital-acquired, while 7/14 (50%) were ventilator-associated. PTX3 was detectable in 22/40 BALfs (55%, mean value 5.66 ± 8.89 ng/ml). Plasma PTX3 was not significantly correlated with BALf PTX3. Circulating PTX3 was not higher when lung infection was present (83.07 ± 126.42 ng/ml vs. 104.7 ± 166.16 ng/ml, *P *= 0.65). At the opposite, PTX3 was more likely to be detectable in culture-positive BALfs in comparison with negative samples (13/14 (93%) vs. 9/26 (34%), *P *= 0.001). The ROC curves analysis showed that alveolar PTX3 was able to diagnose lung infection (AUC = 0.815 (95% CI = 0.675 to 0.954), *P *= 0.001) and that a value of alveolar PTX3 = 0.95 ng/ml predicted pneumonia with 77% specificity and 93% sensitivity.

## Conclusions

BALf PTX3 levels predicted lung infection presence in a relatively large population of critically ill patients. Enrolment of more patients in the present study may disclose the BALf PTX3 role in the diagnosis of pneumonia in the critical care clinical setting.

